# Population-genetic analysis of *HvABCG31* promoter sequence in wild barley (*Hordeum vulgare* ssp. *spontaneum*)

**DOI:** 10.1186/1471-2148-12-188

**Published:** 2012-09-24

**Authors:** Xiaoying Ma, Hanan Sela, Genlin Jiao, Chao Li, Aidong Wang, Mohammad Pourkheirandish, Dmitry Weiner, Shun Sakuma, Tamar Krugman, Eviatar Nevo, Takao Komatsuda, Abraham Korol, Guoxiong Chen

**Affiliations:** 1 Extreme Stress Resistance and Biotechnology Laboratory, Cold and Arid Regions Environmental and Engineering Institute, Chinese Academy of Sciences, Lanzhou 730000, China; 2 Shenzhen Fairy Lake Botanical Garden, Chinese Academy of Sciences, Shenzhen, 518004, China; 3 Institute for Cereal Crops Improvement, Tel Aviv University, Ramat Aviv, Tel Aviv 69978, Israel; 4 National Institute of Agrobiological Sciences, Tsukuba, Ibaraki 305-8602, Japan; 5 Institute of Evolution and Department of Evolutionary & Environmental Biology, University of Haifa, Mount Carmel, Haifa 31905, Israel

**Keywords:** Wild barley, *HvABCG31*, Promoter, Phylogenetic, TFBSs

## Abstract

**Background:**

The cuticle is an important adaptive structure whose origin played a crucial role in the transition of plants from aqueous to terrestrial conditions. *HvABCG31*/*Eibi1* is an ABCG transporter gene, involved in cuticle formation that was recently identified in wild barley (*Hordeum vulgare* ssp. *spontaneum*). To study the genetic variation of *HvABCG31* in different habitats, its 2 kb promoter region was sequenced from 112 wild barley accessions collected from five natural populations from southern and northern Israel. The sites included three mesic and two xeric habitats, and differed in annual rainfall, soil type, and soil water capacity.

**Results:**

Phylogenetic analysis of the aligned *HvABCG31* promoter sequences clustered the majority of accessions (69 out of 71) from the three northern mesic populations into one cluster, while all 21 accessions from the Dead Sea area, a xeric southern population, and two isolated accessions (one from a xeric population at Mitzpe Ramon and one from the xeric ‘African Slope’ of “Evolution Canyon”) formed the second cluster. The southern arid populations included six haplotypes, but they differed from the consensus sequence at a large number of positions, while the northern mesic populations included 15 haplotypes that were, on average, more similar to the consensus sequence. Most of the haplotypes (20 of 22) were unique to a population. Interestingly, higher genetic variation occurred within populations (54.2%) than among populations (45.8%). Analysis of the promoter region detected a large number of transcription factor binding sites: 121–128 and 121–134 sites in the two southern arid populations, and 123–128,125–128, and 123–125 sites in the three northern mesic populations. Three types of TFBSs were significantly enriched: those related to GA (gibberellin), Dof (DNA binding with one finger), and light.

**Conclusions:**

Drought stress and adaptive natural selection may have been important determinants in the observed sequence variation of *HvABCG31* promoter. Abiotic stresses may be involved in the *HvABCG31* gene transcription regulations, generating more protective cuticles in plants under stresses.

## Background

Over their long evolutionary history, plants have evolved morphological, physiological, biochemical, and genetic traits that enable them to respond to abiotic and biotic stresses [1]. Reports on adaptive evolution in plants are numerous, but in general, specific phenotypes associated with putative adaptive events are not well understood [2,3]. One such event, the origin of the cuticle covering their outermost surfaces, was a key adaptation in the transition of plants from aqueous to terrestrial conditions. The cuticle plays an important role in protecting tissue from environmental stresses [4-6] and is especially important for plants growing in drought-prone environments [7]. One of the most important roles of the cuticle is to provide a diffusion barrier against the uncontrolled loss or uptake of water and gases, although it has many other functions such as protection against UV irradiation, mechanical damage, and phytopathogens and herbivorous insects [8]. Among the many genes involved in the process of cuticle formation, *Eibi1* is the only full ABC transporter gene for releasing cuticle compounds reported in cereals. It was identified in wild barley, (*Hordeum spontaneum*), as “*HvABCG31*” by analogy with the rice gene [9,10]. The *HvABCG31* gene was cloned from a spontaneous mutant of wild barley from Israel. This mutant had a drought-hypersensitive phenotype, with a 50% reduction in the major cutin monomers and an incomplete cuticle surface. *HvABCG31* gene was mapped and sequenced and its major function was related to cuticle secretion [10-12].

The distribution centre of *Hordeum vulgare* ssp. *spontaneum,* the progenitor of cultivated barley, lies in the “Fertile Crescent”, starting from Israel and Jordan to southern Turkey, Iraq, Kurdistan, and south-western Iran [13,14], and eastward into Tibet [14]. Israel is located at the junction of three continents and harbours remarkable ecosystemic diversity, such as sharp climatic divisions between northern mesic Mediterranean and the southern xeric desert, topographical diversity from the Dead Sea (the lowest altitude on our planet) to the Golan Heights and Mount Hermon. Environmental heterogeneity is a direct driving force for plant evolution in nature, and wide genetic variation is needed for populations to adapt and survive in highly variable and stressful environments. Domestication and modern plant breeding practices have narrowed the genetic diversity in cultivated plants [15-19]. Extraordinary genetic diversity of wild barley from Israel has been recorded [13,19-21]. Wild barley provides a rich source of potential variation for barley improvement because there are no biological barriers to crossing and meiotic recombination between wild and cultivated barley [13,22,23]. The basic characteristics of barley, such as a short life cycle, few chromosomes (2n = 14), predominant self-pollination, along with a high-density genetic map and high genetic synteny with other Triticeae species, facilitate the exploitation of its potential value for crop improvement.

The coding regions of *HvABCG31* are conserved in monocot and dicot plants [10]. Increasing evidence indicates that the flanking gene regions are highly dynamic [24-26]. Gene sequence variations reflect the genetic and evolutionary history of organisms [27]. Transcription factor binding sites (TFBSs) located at promoter regions are important for regulating gene expression [28].

In this study, the genetic diversity, phylogenetic relationships, and distribution of TFBSs in the *HvABCG31* promoter region were analyzed using 112 wild barley accessions from five natural populations from different habitats in Israel, ranging from the southern xeric Negev desert to the northern mesic Golan Heights. The phylogeny of the *HvABCG31* promoter sequence suggested that adaptation to ecogeographic environmental stresses, including aridity, was an important explanatory factor for the observed variation and history of *HvABCG31*. The analysis of TFBSs in the *HvABCG31* promoter region indicated that gibberellin (GA), light, and abiotic stresses may be involved in the *HvABCG31* transcriptional regulation.

## Results

### Genetic analysis of the *HvABCG31* promoter sequence from five *H. vulgare* ssp. *spontaneum* populations in Israel

**Figure 1:**
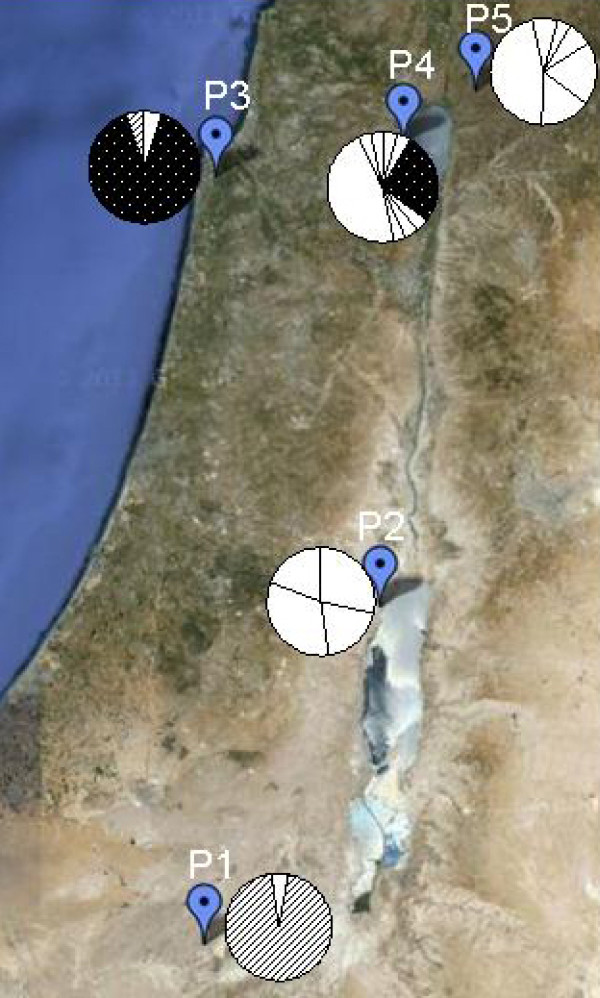
**Haplotype distribution in five natural populations of *Hordeum vulgare *ssp. s (*Spontaneum*) in Israel.** The white sectors in the pies indicate the frequencies of haplotypes unique to that population, while patterned sectors denote the frequencies of haplotypes shared between populations (for frequency details, see Table 2). P1, Mitzpe Ramon; P2, Dead Sea; P3, "Evolution Canyon"; P4, Arbel; P5, Yehudiyya.

Sequences of the *HvABCG31* promoter were compared among 112 wild barley accessions from five natural populations: Mitzpe Ramon (P1; southern Israel), Dead Sea (P2; southern), "Evolution Canyon" (P3; northern), Arbel (P4; northern), and Yehudiyya (P5; northern) (Figure 1; Table 1). The total length of the aligned *HvABCG31* promoter sequences was 2148 bp. We identified 22 haplotypes; all haplotype sequences were deposited in the DDBJ database ( http://sakura.ddbj.nig.ac.jp/top-e.html) with the accession numbers AB709909–AB709930. Over 90% of the haplotypes were unique to a population: 20 haplotypes were population-specific, while only two were shared between populations in this study (Figure 1; Table 2).

**Table 1:** **Description of the natural wild barley (*Hordeum vulgare *ssp. *spontaneum*) populations sampled in this study**

**Population**	**Number of accessions**	**Location**	**Latitude (N) Longitude(E)**	**Altitude(m)**	**Mean annual rainfall(mm)**	**Mean January temperature(°C)**	**Minimal January temperature(°C)**	**Annual evaporation(cm)**	**Soil type**
P1	20	Mitzpe Ramon	30°37'26.6" 34°50'22.4"	519	69.4	9.8	2.3	240	Desert alluvium
P2	21	Dead Sea	31°43'15.8" 35°27'05.3"	−390	97.7	14	4.8	260	Desert alluvium
P3	19	“Evolution Canyon”	32°42'48" 34°58'34"	61	621.8	11.8	2.5	180	Terra rossa
P4	26	Arbel	32°49'19.0" 35°29'43.2"	133	448.7	10.5	2.2	230	Terra rossa
P5	26	Yehudiyya	32°56'07.6" 35°41'50.3"	172	521.1	9.9	2.1	230	Basaltic

**Table 2:** **Haplotype frequencies in five Israeli natural population of wild barley (*Hordeum vulgare *ssp. *spontaneum*)**

	**P1 (20)**	**P2 (21)**	**P3 (19)**	**P4 (26)**	**P5 (26)**
Hap 1	0.95 (19)	0	0.0526(ES) (1)	0	0
Hap 2	0.05 (1)	0	0	0	0
Hap 3	0	0.286 (6)	0	0	0
Hap 4	0	0.19 (4)	0	0	0
Hap 5	0	0.333 (7)	0	0	0
Hap 6	0	0.19 (4)	0	0	0
Hap 7	0	0	0.895(ES + AS) (17)	0.269 (7)	0
Hap 8	0	0	0.0526(AS) (1)	0	0
Hap 9	0	0	0	0.0385 (1)	0
Hap 10	0	0	0	0.0385 (1)	0
Hap 11	0	0	0	0.0385 (1)	0
Hap 12	0	0	0	0.462 (12)	0
Hap 13	0	0	0	0.0385 (1)	0
Hap 14	0	0	0	0.0385 (1)	0
Hap 15	0	0	0	0.0385 (1)	0
Hap 16	0	0	0	0.0385 (1)	0
Hap 17	0	0	0	0	0.0769 (2)
Hap 18	0	0	0	0	0.192 (5)
Hap 19	0	0	0	0	0.154 (4)
Hap 20	0	0	0	0	0.462 (12)
Hap 21	0	0	0	0	0.0769 (2)
Hap 22	0	0	0	0	0.0385 (1)

Note: ES refers to ‘European Slope’ and AS refers to ‘African Slope’ of “Evolution Canyon”, Lower Nahal Oren, Mount Carmel [31]; The number in the bracket refers to the number of accessions in each population.

Based on the major environmental factors of average rainfall and soil type (Table 1), the five populations could be divided into the southern xeric group (P1 and P2) and the northern mesic group (P3, P4, and P5). The northern mesic group included 17 haplotypes, most with low frequencies; whereas six haplotypes were found in the southern xeric group. Four of five populations harboured a population-specific high-frequency haplotype; the exception, P2, comprised several haplotypes in similar proportions (Figure 1). In population P1, the majority of accessions (19 of 20) had one haplotype (Hap 1), and in population P3, 17 of 19 accessions shared one haplotype (Hap 7). Interestingly, no haplotypes were shared between P1 and P2 (southern) or between P4 and P5 (northern). Two haplotypes were shared between populations: Hap 7 occurred in most accessions (17 of 19) of P3 and in some accessions in P4, which has the same soil type (terra rossa); Hap 1 was shared in P3 and P1, although the P3 accession was an isolated individual from the ‘European slope’ of “Evolution Canyon”.

The genetic variation within and between populations was calculated by AMOVA. The genetic divergences were remarkably high (Table 3). Interestingly, more genetic variation occurred within populations than among populations (54.2% and 45.8%, respectively); the estimated Fst was.

**Table 3:** Analysis of molecular variance (AMOVA)

**Source of variation**	**d.f.**	**Sum of squares**	**Variance components**	**Percentage of variation**
Among groups	1	5.913	0.022 Va	4.4
Among populations within groups	3	14.729	0.207 Vb	41.4
Within populations	107	28.929	0.270 Vc	54.2
Total	111	49.571	0.499	

The genetic diversity analysis and the neutrality test results in different populations are summarized in Table 4. There were more haplotypes, and greater haplotype diversity (Hd) in two northern populations (P4 and P5), but the highest Hd and nucleotide diversity (π) were observed in P2 (Dead Sea). Two neutrality tests (Tajima, and Fu & Li tests) showed that different populations experienced different selection pressures. No significant evidence for selection was identified in P4 and P5, which together harboured 15 haplotypes. Significant positive values were obtained for P2 using both the Tajima and Fu & Li tests, suggesting balancing selection on the *HvABCG31* promoter region in this population. In contrast, significant negative values for both tests were obtained for P1 and P3, which could have been caused by purifying selection, demographic effects (i.e., population expansion), or low frequencies of harmful mutations [2,29,30]. However, despite predominant haplotypes, low-frequency haplotypes were found in both P1 and P3 (Figure 1; Table 2). One accession from P1 had Hap 2, and two accessions from P3 had Hap 1 and Hap 8. 

**Table 4:** Genetic diversity and neutrality test

	**P1**	**P2**	**P3**	**P4**	**P5**	**Total**
Number of accessions	20	21	19	26	26	112
Number of haplotypes, h	2	4	3	9	6	22
Haplotype diversity, Hd	0.100	0.771	0.205	0.732	0.742	0.893
Nucleotide diversity, π	0.001	0.009	0.002	0.0004	0.001	0.006
Tajima's D test	−2.452 ***	2.244 *	−2.278 **	−1.062	−1.119	−0.553
Fu and Li's F* test	−3.917 **	2.179 **	−3.068 **	−2.176	0.567	−0.133

Statistical significance in Tajima's D test: * P < 0.05, ** P < 0.01, *** P < 0.001; Statistical significance in Fu & Li's F* test: **P < 0.02.

### Phylogenetic analysis of *H. vulgare ssp. spontaneum HvABCG31* promoter sequence

**Figure 2:**
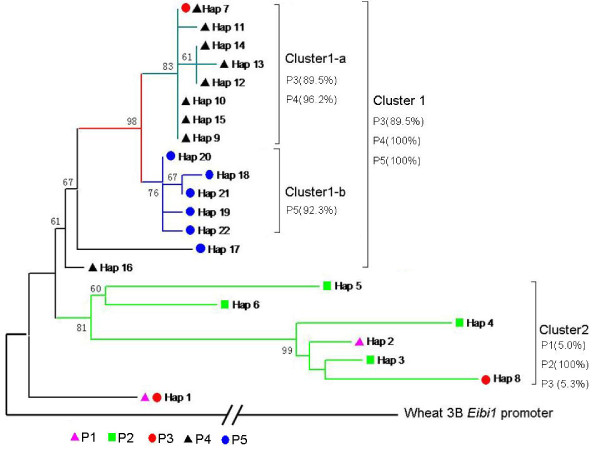
**Maximum likelihood bootstrap consensus phylogeny of 2 kb sequence of the *HvABCG31 *promoter.** The phylogenetic consensus tree was constructed using the method of maximum likelihood in MEGA5. Values at the nodes are bootstrap percentage > 60%. Percentages below the cluster names refer to the accession proportions in specific populations. P1, Mitzpe Ramon; P2, Dead Sea; P3, "Evolution Canyon"; P4, Arbel; P5, Yehudiyya.

To investigate the relationships among the different populations, a phylogenetic tree based on SNP haplotypes was constructed using maximum likelihood as implemented in MEGA5 software (Figure 2). The tree was rooted with the promoter sequence of a putative *HvABCG31* homologue from the wheat (*Triticum aestivum* var. Chinese Spring) 3B chromosome. Most accessions (69/71 = 97.2%) from the three northern mesic populations (from 89.5% of P3, 100% of P4, and 100% of P5) formed one cluster (Cluster 1). This cluster was further divided into two major sub-clusters with high support values: Cluster 1-a from P3 and P4 and Cluster 1-b from P5. Interestingly, the structure of the sub-clusters was in accordance with the soil types of their collection sites (terra rossa in Cluster 1-a; basalt in Cluster 1-b). The second cluster (Cluster 2) included all 21 accessions from P2, one accession from the xeric population of P1, and one accession from the xeric ‘African Slope’ of P3. One shared haplotype (Hap 1) from both P1 and P3 proved very distant from both clusters and represented most of the accessions from P1 (19/20 = 95%) and one accession from the ‘European Slope’ of P3.

**Figure 3:**
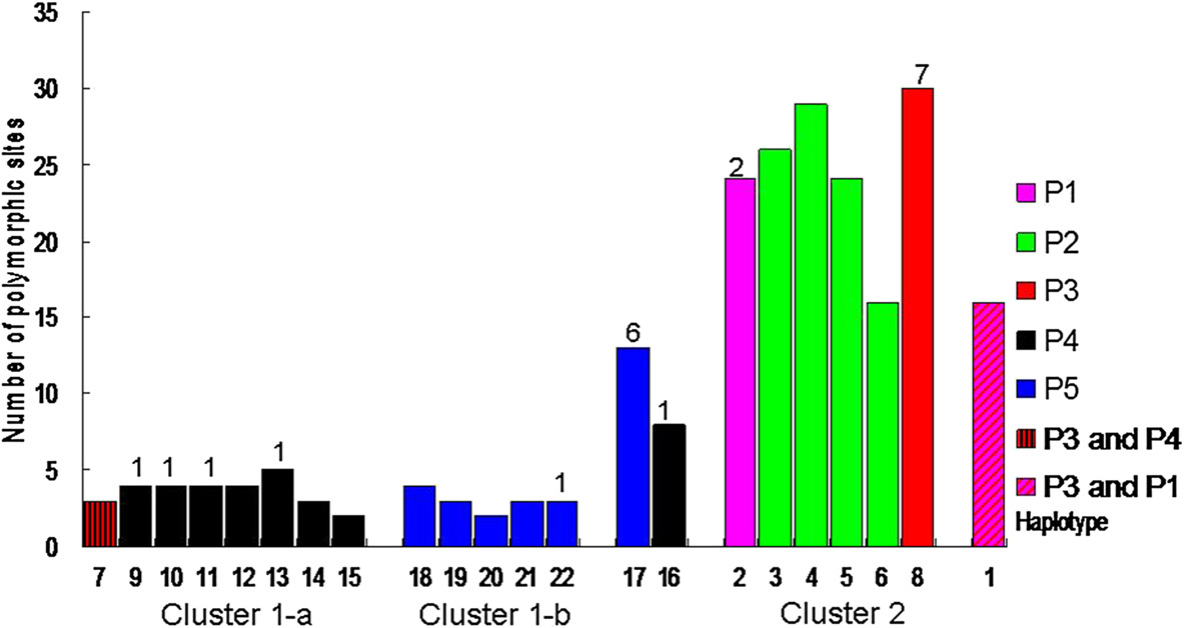
**Differences between haplotypes and the consensus sequence of *HvABCG31 *promoter in *Hordeum vulgare *ssp*. spontaneum.*** The number of rare polymorphic sites for each haplotype are above the bars. P1, Mitzpe Ramon; P2, Dead Sea; P3, "Evolution Canyon"; P4, Arbel; P5, Yehudiyya.

The distances among the haplotypes in Cluster 1 (including Clusters 1-a and 1-b) were closer than those in Cluster 2 (see Figures 2, 3). Figure 3 depicts this pattern in more detail. The total number of polymorphic sites (90) was determined from the alignment of all 22 haplotypes (see Additional file 1: Table S1). The number of differences between each of the haplotypes and the consensus sequence varied from 2–30. There were 15 haplotypes in Cluster 1 and six in Cluster 2; one haplotype (Hap 1) was separated from all others. The majority of haplotypes (13 of 15) in Cluster 1 differed by 2–5 bases from the consensus sequence, with the exceptions of Hap 16 (8 differences) and Hap 17 (13 differences). In Cluster 2, haplotypes differed from the consensus sequence by 16–30 bases, and the isolated Hap 1 had 16 differences. A total of 21 rare polymorphisms were scattered among different haplotypes represented by one or two accessions. Rare sites were found in about half of the haplotypes (5/9 = 55.6%) from P4, in one haplotype from each P1 and P3, and in two haplotypes from P5. Most of the rare sites occurred in a few haplotypes, like Hap 17 from P5 (with 6 rare sites) and Hap 8 from P3 (with 7 rare sites). Most of the rare polymorphic sites (19 of 21) appeared in three northern mesic populations (P3, P4, and P5). Southern arid populations had fewer haplotypes, but they differed from the consensus sequence at a large number of positions, while northern mesic populations had more haplotypes that were, on average, closer to the consensus sequence.

### Transcription factor binding sites (TFBSs) in the *HvABCG31* promoter

**Figure 4:**
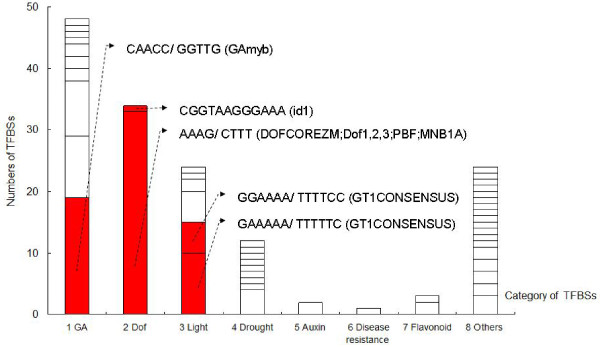
**Numbers and categories of transcription factor binding sites (TFBSs) in the *HvABCG31 *promoter of *Hordeum vulgare *ssp*. spontaneum*.** Different grids in the histogram refer to specific transcription factor binding sites; Sites indicated in red occurred at significantly higher frequencies than random, based on a permutation test with 10,000 runs; their sequences are given and related transcription factors are listed in brackets.

The total number of TFBSs was 148 with a range of 121–134 among the haplotypes. There were 121–128 (P1) and 121–134 (P2) TFBSs in the two southern arid populations, and 123–128 (P3), 125–128 (P4), and 123–125 (P5) sites in the three northern mesic populations. The TFBSs could be classified into 8 categories according to their basic functions (see Additional files 2 and 3: Table S2 and Table S3). Because the TFBSs were rather short (4–12 bases) and could have occurred randomly, their presence in the promoter sequences were statistically analyzed using a permutation test (10,000 runs). A few TFBSs related to GA, Dof, and light were significant (Figure 4; red). Many binding sequences related to various functions occurred at low frequency and were not significant. Most were singletons or duplets, but some more common ones for GA (9–10 copies) and light (5 copies) also proved to be non-significant. One singleton with a long binding sequence (12 bp) proved statistically significant (see Figure 4).

**Figure 5:**
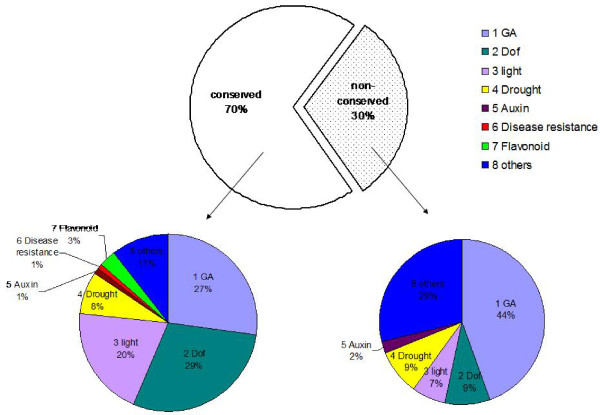
**Proportions of conserved and non-conserved TFBSs in the *Hordeum vulgare *ssp*. spontaneum HvABCG31 *promoter sequence.** The TFBSs were analysed using PlantPan database ( http://plantpan.mbc.nctu.edu.tw/index.php). The upper pie chart showed the over all proportions of conserved and non-conserved TFBSs, and the two lower pie charts showed the proportions of different categories of TFBSs within each group.

**Figure 6:**
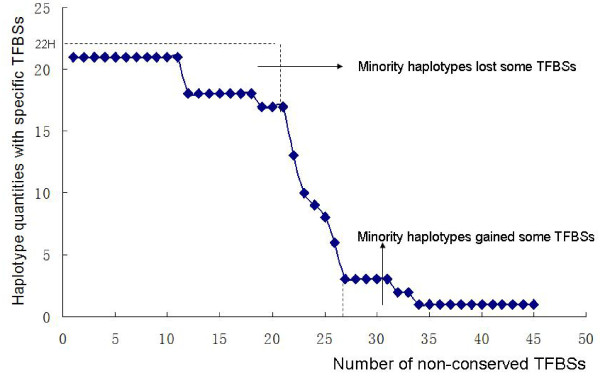
**Distribution of non-conserved TFBSs in all 22 haplotypes of the *HvABCG31 *promoter.** The upper dashed area refer to TFBSs that occurred in a majority of haplotypes, and the lower dashed area refer to those occurring in a minority of haplotypes.

Of the 148 TFBSs, about 70% were located in the conserved regions of the 22 haplotypes, whereas the rest appeared in the non-conserved regions (Figure 5, upper pie chart). Interestingly, the conserved regions included a high proportion of TFBSs related to GA, Dof, and light (Figure 5, bottom left). In contrast, in the non-conserved regions, the GA TFBSs signals were predominant (44%) (Figure 5, bottom right). For example: among the 45 TFBSs in non-conserved regions, 20 were related to GA. Figure 6 shows the distribution of non-conserved TFBSs in all 22 haplotypes: of the 45 non-conserved TFBSs, 21 were absent in the minority haplotypes (Figure 6, upper dash area), while 19 appeared in some minor haplotypes (Figure 6, bottom dash area). A high proportion of non-conserved TFBSs (40/45 = 88.9%) affected minor haplotypes. Most of these minor haplotypes (bold and italic numbers in Additional file 2: Table S2) were related to haplotypes 1–6, 8 in 43 accessions; most of these accessions (41/43 = 95.3%) originated from southern xeric populations (P1 and P2); the other two accessions came from the two slopes (‘African Slope’ and ‘European Slope’) of P3.

## Discussion

### Genetic differentiation of *HvABCG31* promoter sequences

High genetic diversity was found in the *HvABCG31* promoter region (Hd = 0.893; π = 0.006) (Table 4) in the present study. Similar results for the *Dehydrin 1* promoter region (Hd = 0.946; π = 0.0054) were reported in a study of wild barley populations from Israel [31]. Numerous studies at global, regional, and local scales have demonstrated that genetic diversity is associated with stressful environments. In wild barley, this was shown using allozymes and DNA markers (RAPDs, SSRs, AFLPs, and rDNA) [32-36]. Drought is a major evolutionary driving force affecting plant genetic diversity and population genetic structure [37,38]. The sharp climatic division between the northern mesic Mediterranean region and southern xeric Negev Desert in Israel generates a gradient of increasing aridity. Our phylogenetic analysis of *HvABCG31* promoter sequences grouped most accessions (69/71 = 97.2%) of the northern populations (P3, P4, and P5) into one cluster, whereas all accessions from the Dead Sea (P2), one accession from Mitzpe Ramon (P1), and one accession from the ‘African Slope’ of “Evolution Canyon” (P3) formed a second cluster. Interestingly, there were many indel polymorphisms in our five natural populations but the basic phylogenetic structure was similar whether or not indels were considered (results not shown). The major ecological factors differentiating the northern (P3, P4, and P5) and southern (P1 and P2) populations, excluding geographic distance, were average annual rainfall and soil type. Water availability is the most important environmental factor for plant growth [22,39]. Drought stress is influenced by several factors including soil type, rainfall, temperature, and rate of evaporation. Interestingly, the sub-clusters in Cluster 1 were characterized by different soil types, terra rossa in Cluster 1-a and basalt in Cluster 1-b. Therefore, drought stress may explain this separation [40-42]; however, we cannot exclude the influence of other factors, such as isolation by distance.

In the current study significant evidence against neutrality (based on Tajima's D test and Fu & Li's F* test) was found in the two southern populations (P1 and P2) and in one northern populations (P3), while no significant deviations from neutrality were found in the other two northern populations (P4 and P5). Significant positive values of the test statistics indicate balancing selection, while significant negative values may suggest purifying selection and demographic effects (population expansion) [2,29,30,43-45]. Balancing selection detected in population P2 may relate to environmental heterogeneity. Our Mitzpe Ramon (P1) population is located in stressful habitats in the Negev Desert, and P2 is located in the Dead Sea area, with highly heterogeneous and stressful conditions including severe drought and salinity stresses. “Evolution Canyon” (P3) consist of two opposite slopes: the warm-dry ‘African Slope’ is drastically divergent from the cool and humid ‘European Slope’, although they are separated by an average distance of only 200 meters. Much research on “Evolution Canyon” has proved that the interslope divergent selection is due to microclimate [46,47] (see the publication list: http://evolution.haifa.ac.il/index.php/component/content/article/29).Different selection pressures in different regions are important in adaptation to local habitats [48,49]. Our results allowed us to assume that different selection pressures in our five populations affected adaptation to different environments. Water availability may be an important selection pressure [38]. However, more extensive sampling is needed to support the hypothesis that polymorphisms in the *HvABCG31* promoter are maintained by variable drought and salinity.

### *HvABCG31* expression may be regulated by transcription factors related to abiotic stress

Although *HvABCG31* is involved in cuticle formation [10], only three TFBSs related to flavonoid biosynthesis were found in its promoter region. TFBSs copy numbers are considered to be important for transcriptional regulation [50]. The TFBSs related to three factors (GA, Dof, and light) were significantly enriched in the *HvABCG31* promoter. Previous studies showed that cuticle formation is affected by drought and light [51-53]. The Dof proteins are a family of plant-specific transcription factors involved in the control of multiple functions in plant growth and development including stress, light, and plant hormone responses [54]. GA is an important plant hormone that controls diverse aspects of growth and development [55]. In the non-conserved regions of the *HvABCG31* promoter, most base changes (44%) added or deleted GA-binding sites. Of note, a recent microarray analysis of drought-stressed wild emmer wheat roots indicated that GA may be involved in drought resistance [56]. In addition, GA plays a crucial role in barley protection under drought and other stresses [57]. GA, light, and abiotic stresses may affect *HvABCG31* transcription, which may indirectly indicate that *HvABCG31* affects abiotic stress tolerance, although other processes may influence *HvABCG31*.

Base variation can dramatically change TFBSs and thus affect gene regulation [28]. We found 45 non-conserved TFBSs, some of which were lost or gained in the *HvABCG31* promoters of minor haplotypes, mainly in the southern (xeric) populations. An early paper reported that TFBSs can appear or disappear among closely related species and even within populations [26]. Nevertheless, poor correlation exists between the divergence of TFBS sequences and gene expression, suggesting that gene expression might be regulated by compensatory mechanisms [58]. Loss of binding sites may be buffered by the presence of other binding sites for additional factors that are involved in the same process or that bind cooperatively with the factor whose binding site has diverged [59]. It remains unknown whether such compensatory mechanisms have evolved in the control of barley *HvABCG31*; more expression experiments are required to further understand its regulation.

## Conclusions

Our phylogenetic analysis of eco-geographical variation in the 2kb promoter region of the *HvABCG31* gene in five natural populations of *H. vulgare* ssp. *spontaneum* in Israel suggests that aridity may be an important factor affecting the observed sequence variation*.* The analysis of TFBSs in the *HvABCG31* promoter region indicated that GA, light, and abiotic stresses may be involved in the *HvABCG31* transcription regulation and that *HvABCG31* may be involved in abiotic stress responses, both locally and regionally, thereby generating adaptive structures in response to environmental stresses.

## Methods

### Plant materials

Seeds of 112 accessions of *H. vulgare ssp. spontaneum* were collected from five natural populations in Israel during April 2007. The seeds were collected randomly from individuals situated at least 1–2 m apart [31]. We analyzed the genotypes of 19 to 26 accessions per population. The five collection sites were characterized by distinct environmental factors (Table 1). The two sites in southern Israel were (P1) Mitzpe Ramon, located in the Negev Desert and (P2) the Dead Sea area, known for its highly saline and arid environment. In northern Israel, “Evolution Canyon” (P3), at Lower Nahal Oren, Mount Carmel, near the Mediterranean coast, is a designated research microsite harbouring two distinct habitats (cool-mesic and warm-xeric) with an average distance of 200 m [32,46,47,60]. Plants were collected from similar altitudes but different slopes: 11 from the xeric south facing slope designated as ‘African Slope’ and 8 from the mesic northern facing slope, designated as ‘European Slope’; The final two populations in northern Israel were (P4) Arbel mountain, close to the Sea of Galilee; and (P5) Yehudiyya, located in the Golan Heights. The latitude, longitude, and altitude data indicated in Table 1 were recorded by a GPS (Global Positioning System) receiver, and soil types were recorded when sampled in the fields. The climatic data (average rainfall, temperatures in the barley growth season, and evaporation rates) were obtained from the BioGIS-Israel biodiversity web site ( http://www.biogis.huji.ac.il/Map.aspx). All the data were checked using a relevant reference [61] and the records of the nearest meteorological stations from 1980–2009 (provided by the Israeli Meteorological Service: http://ims.gov.il/IMS/CLIMATE).

### DNA extraction, amplification, and sequencing

Seeds were germinated in Petri dishes on filter paper with purified water, treated with cold (4°C) for 7 d then held at 25°C for 3 d. Seedlings were then transplanted into pots (16 cm high, 18 cm diameter) with a cycle of 16 h, 22°C day and 8 h, 18°C night. Genomic DNA was extracted from young leaves using a small-scale isolation method [62]. Based on our previous studies of *HvABCG31*[11,12], one pair of primers was designed to amplify the gene promoter region: EIBI1antiR13678: TGAGCAAAGGAGCAAGGA and ABCcontig7F1550: CGGGGAGCAAAGAAAATGTA. Four single primers were used to sequence the promoter: ABCcontig7F1767: ACTACGGGCGACCTGAGCA; EIBI1F11844: TCTTTGATCGTTGGGGTTTT; EIBI1F12436: GTGCCCCGTATTGTTCTCAT and EIBI1antiF13068: AGATTTTCCACCATGCCTGT. All primers were designed using Primer3 ( http://frodo.wi.mit.edu/primer3/).

The PCR amplifications were carried out in a C1000TM thermal cycler (Bio-Rad, Hercules, CA, USA). Each 50 μL PCR reaction contained 20 ng template DNA, 5 μL 10× PCR buffer, 1.25 U ExTaq DNA polymerase (Takara, Beijing, China), 5 μL MgCl_2_ (25 mM), 4 μL dNTP (2.5 uM), 1.5 μL each primer (10 uM), 3 μL DMSO (Dimethyl Sulfoxide, Tianjin, China) and water. The PCR procedure consisted of an initial denaturing step (94°C, 5 min); followed by 30 cycles of 94°C for 1 min, 60°C for 1 min, and 72°C for 2 min, and completed by an incubation at 72°C for 15 min. Amplified DNA products were electrophoresed on 1–2% w/v agarose gels and sequenced on an ABI 3100 DNA sequencer (Applied Biosystems, Foster City, CA, USA).

### DNA sequences analysis

Overlapping DNA sequences were assembled using DNAMAN6.0 ( http://en.bio-soft.net/format/DNAMAN.html), and ambiguous sites were checked manually in accordance with the DNA peak files in Chromas2.01 ( http://www.technelysium.com.au/chromas_lite.html). Sequences were aligned with the MUSCLE feature in MEGA5 ( http://www.megasoftware.net/) [63,64]. Genetic diversity, haplotype identification, and neutrality tests (Tajima tests and Fu & Li tests) were conducted in DnaSP5.10 ( http://www.ub.edu/dnasp/). Gaps were considered a fifth state [65]. Haplotypes occurring in a single population were considered to be unique, and in more than one population were considered shared. An analysis of molecular variance (AMOVA) was performed in Arlequin 3.1 ( http://cmpg.unibe.ch/software/arlequin3/) based on the haplotype frequencies [66]. Genetic differentiation was evaluated by the F statistics, Fst [67,68]. Polymorphic sites were determined manually based on an alignment of all haplotypes, and each haplotype was characterized by the number of sites by which it differed from the consensus sequence [69,70]. An allele was considered to be rare in fewer than 2% (1–2 accessions) in this study. Tandem gaps were treated as a single mutant event (insertion or deletion) and were therefore counted as single polymorphic sites. Each polymorphic (polymorphic) site was considered an independent unit of variation in the analysis [71].

A phylogenetic tree of haplotypes sequences was constructed using the maximum likelihood (ML) algorithm in MEGA5, with 1000 bootstrap replicates to gauge support, and a 60% cut off was used in the analysis. The phylogenetic tree was rooted using 2 kb of the putative homologue from wheat which was obtained from a BLAST search of the 3B chromosome sequences (kindly provided by Frédéric Choulet, INRA; Clermont Ferrand, France). All haplotypes of the *H. vulgare* ssp. *spontaneum HvABCG31* promoter were scanned for TFBSs using the PlantPan database ( http://plantpan.mbc.nctu.edu.tw/index.php), which combines several plant TFBS databases, such as PLACE, TRANSFAC, JASPER, and AGRIS [72]. The search was conducted using the monocots transcription factor libraries (wheat, barley, maize, and rice) because distinct differences in the evolution of an upstream region exist between monocots and dicots [73]. A special script was developed to test the significance of highly abundant binding signals of specific TFs. Using this program, the original DNA sequences were reshuffled to produce randomized sequences, which were scored for the occurrence of specific TFBS signals that proved abundant in our original sequences. Using 10,000 such permutation runs, we evaluated the statistical significance of these original binding signals.

## Competing interests

The authors declare that they have no competing interests.

## Authors’ contributions

XM, GJ, and GC had the main responsibility for the study and participated in all aspects, including developing the main idea, hypotheses, setting up the study, collecting some of the data, analyzing the data, and preparing and revising the manuscript. CL, AW, MP, SS, and TK carried out the molecular genetic studies, including DNA extraction, sequencing and data analysis. HS, DW, TK, XM, EN, and AK interpreted data and helped to revise the manuscript. All authors read and approved the final manuscript.

## Supplementary Material

Additional file 1 **Table S1.** Detailed information for the polymorphic sites sample. Click here for file

Additional file 2 **Table S2.** Detailed information for 22 haplotype in wild barley (*Hordeum vulgare* ssp. *spontaneum*). Click here for file

Additional file 3 **Table S3.** Detailed information for TFBSs classification. Click here for file
